# Strain Amount Dependent Grain Size and Orientation Developments during Hot Compression of a Polycrystalline Nickel Based Superalloy

**DOI:** 10.3390/ma10020161

**Published:** 2017-02-10

**Authors:** Guoai He, Liming Tan, Feng Liu, Lan Huang, Zaiwang Huang, Liang Jiang

**Affiliations:** 1 State Key Laboratory of Powder Metallurgy, Central South University, Changsha 410083, China; heguoai@csu.edu.cn (G.H.); limingtan@csu.edu.cn (L.T.); liufengehe@csu.edu.cn (F.L.); lhuang@csu.edu.cn (L.H.); liangjiang@csu.edu.cn (L.J.); 2 Powder Metallurgy Research Institute, Central South University, Changsha 410083, China; 3 High Temperature Materials Research Institute, Central South University, Changsha 410083, China

**Keywords:** nickel base superalloy, hot deformation, high-throughput experimental method, double cone, misorientation, grain size

## Abstract

Controlling grain size in polycrystalline nickel base superalloy is vital for obtaining required mechanical properties. Typically, a uniform and fine grain size is required throughout forging process to realize the superplastic deformation. Strain amount occupied a dominant position in manipulating the dynamic recrystallization (DRX) process and regulating the grain size of the alloy during hot forging. In this article, the high-throughput double cone specimen was introduced to yield wide-range strain in a single sample. Continuous variations of effective strain ranging from 0.23 to 1.65 across the whole sample were achieved after reaching a height reduction of 70%. Grain size is measured to be decreased from the edge to the center of specimen with increase of effective strain. Small misorientation tended to generate near the grain boundaries, which was manifested as piled-up dislocation in micromechanics. After the dislocation density reached a critical value, DRX progress would be initiated at higher deformation region, leading to the refinement of grain size. During this process, the transformations from low angle grain boundaries (LAGBs) to high angle grain boundaries (HAGBs) and from subgrains to DRX grains are found to occur. After the accomplishment of DRX progress, the neonatal grains are presented as having similar orientation inside the grain boundary.

## 1. Introduction

Powder metallurgy nickel base superalloys have been extensively applied in rotating parts of aero turbine engines due to their exceptional mechanical properties and trustworthy corrosion resistance at elevated temperatures [1,2,3]. The superb mechanical performance is closely related to grain size and precipitate size/distribution/volume fraction. Generally, the in-service components with uniform and fine grains are required and fabricated via a series of processes including powder atomization, hot isostatic pressing, hot extrusion, and isothermal forging. To realize near-net-shaping of the industry-scale product, the capability of superplastic deformation is required and the grain size needs to be carefully controlled throughout hot forging. Previous results [4,5,6] showed that dynamic recrystallization (DRX) occupied the dominant position in refining the grain size and promoted superplastic forming. The DRX process was documented as being closely related to temperature [7] and strain rate [8,9], and was usually initiated when the strain reached a critical value [10,11,12]. To date, previous reports have widely investigated the effects of deformation temperature, loading strain rate and strain amount on grain size evolution mostly using hot compression of cylindrical specimens [13,14,15,16,17]. The strain amount was proven to control the initiation of DRX progress under given deformation temperature and strain rate. On the other hand, too large an amount of height reduction was more inclined to induce cracking [3], in particular under low temperatures and high stain rates. The value of critical strain was usually determined by mathematical calculation from strain-stress curves or microstructure observation. However, these techniques require a large number of specimens to realize different height reductions. In addition, it was indicated that strain gradients existed randomly in the cylindrical specimen by simulation and experiment [18], which alters with variation of the end friction and is difficult to measure. Recently, the double-cone specimen provided a new perspective to study the effect of strain on microstructure characteristics. Wherein, a wide range of strain that distributed from the edge to the center of specimen can be achieved after deformation. By far, the double cone specimen was mostly utilized to study the abnormal grain growth [19], recrystallization behavior [20,21,22], and simulate the grain size evolution [23,24,25] in hot deformation of nickel base superalloy.

In this article, we employed the double cone specimen to investigate the grain size evolution of a newly designed PM nickel base superalloy. The microstructure developments were characterized using the techniques of EBSD and finite element method. The orientation, grain boundaries evolution, and grain size are measured to bridge the relationship with strain amount. Simulation results show that the impacts of interfacial friction on strain distribution across the double cone specimen are proven to be inconsequential. The strain amount is experimentally observed to have considerable influence on grain size and the characteristics of grain boundaries. Accordingly, the effects of strain amount on DRX grain size concerning initial grain size and deformation conditions are discussed. Through introducing the high-throughput double cone specimen into the hot working process, the investigation period and also the number of samples required can be reduced substantially. Our results provide a new sight in understanding the effects of strain amount on microstructure evolution by high-throughput experiments and expect to trigger broad interests in the hot-working field. 

## 2. Materials and Experimental Procedures

The chemical composition of the newly designed superalloy used in the present work is summarized in Table 1. The master alloy was prepared by means of vacuum induction melting, which was then subjected to produce the nearly spherical powder via plasma rotating electrode process. The powder was subsequently screened and loaded into a stainless steel container, which was then heated to about 400 °C and vacuum degassed to 10^−3^ Pa for 24 h before it was sealed. Hot isostatic pressing (HIP) was applied to consolidate the powder for 4 h under the conditions of 1100 °C and 140 MPa. Following by HIP consolidation, the superalloy billet was cut out and re-caned for hot extrusion to an area reduction of 10:1 at temperature of 1100 °C.

**Figure 1:**
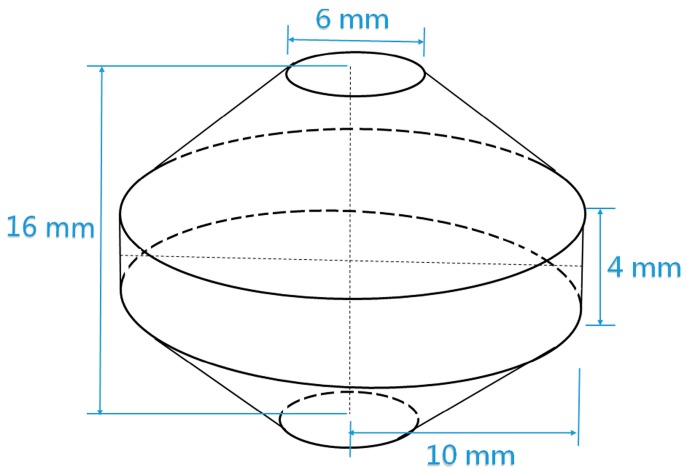
Illustration of the shape and dimensions of double cone specimen.

Dimensions of the double cone specimen (DCS) are schematically shown in Figure 1. A long bar was cut out from the center of the extruded billet and machined precisely to the exact sizes of the double cone. Prior to deformation, all the DCSs were subjected to heat treatment at 1050 °C for 1 h to reduce the dislocation density. After that, isothermal compressions were carried out on a MTS machine (MTS 810.13, Minneapolis, USA) at 1100 °C with height reduction rate of 0.0093 mm·s^−1^. The deformed specimens were cut in half parallel to the compression direction by a low-speed diamond saw. After that, the sections were prepared by standard metallographic techniques and finished by polishing with 0.05 μm colloidal silica to remove the surface scratches. In order to get a high solution in EBSD observation, vibration polishing was carried out for 8 h. For TEM observation, discs with a thickness of 70~80 μm were twin-jet electro polished in a corrosive solution of ethanol and perchloric acid (9:1 in volume fraction) at a temperature of −25 °C and a voltage of 20 V. A field-emission SEM (FEI Quanta 650) which is equipped with an EBSD detector and Channel 5 software was employed to examine the boundaries evolutions and determine the grain size. To optimize the diffraction patterns, an acceleration voltage of 20 kV, step size of 0.15 μm, and spot size of 5.0 were employed. All the parameters were kept consistent throughout each scan. Misorientation of 15° was applied to discriminate the high angle grain boundaries (HAGBs) and low angle grain boundaries (LAGBs). The average grain sizes were determined by calculating the average equivalent circle diameter, and the corresponding count/area fraction was also obtained. TEM observation was carried out on a field-emission TEM (Tecnai G2 F20, FEI) under the accelerating voltage of 200 kV.

The compression process was simulated by employing finite element method software Deform 3D simulation package (SFTC, Ohio State, USA). This software was widely utilized to predict the strain level and its distribution inside the deformed parts. To simulate the compression process more precisely, the corresponding material properties were obtained from previous research on the alloy. Due to the relatively low deformation rate, the temperature rise driven by deformation could be ignored.

## 3. Results and Discussion

### 3.1. Pristine Microstructure 

**Figure 2:**
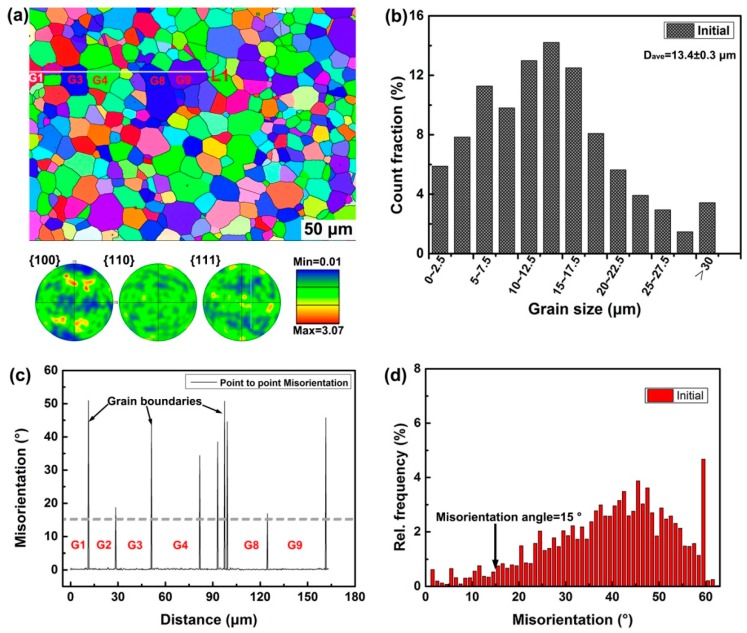
Illustration of pristine microstructure observations for as-extruded alloy after heat treatment: (**a**) The IPF color map reveals that the as-extruded alloy consists of equiaxed grains with very weak crystallographic texture (inset); (**b**) Statistical results of grain size distribution indicating the uniform and fine grains with an average grain size of 13.4 ± 0.3 μm; (**c**) Detection of misorientation shows the orientations within the grains are very similar to each other; (**d**) Grain boundary misorienations distribution, indicating nearly recrystallized microstructure is obtained.

Figure 2 demonstrates the microstructure observations of as-extruded alloy after heat treatment. The inverse pole figure (IPF) color map shown in Figure 2a reveals that the as-extruded alloy consists of equiaxed grains with very weak crystallographic texture (inset). Further statistical analyses indicate the uniform and fine microstructure with an average grain size of 13.4 ± 0.3 μm is achieved after hot extrusion. Detection of misorientation along line 1 (G1, G2,… and G9 represent nine grains detected) shows the orientations within the grains are very similar to each other, while the grain boundaries are present as HAGBs, as shown in Figure 2c. The identical orientation within a specific grain indicates a ‘clean’ structure with low dislocation density is achieved after annealing treatment. Through overall analysis, up to 95% of grain boundary misorienations belong to the HAGBs (Figure 2d), indicating nearly recrystallized architectures are obtained after hot extrusion and annealing. 

### 3.2. Deformation Modeling and Strain Distribution 

**Figure 3:**
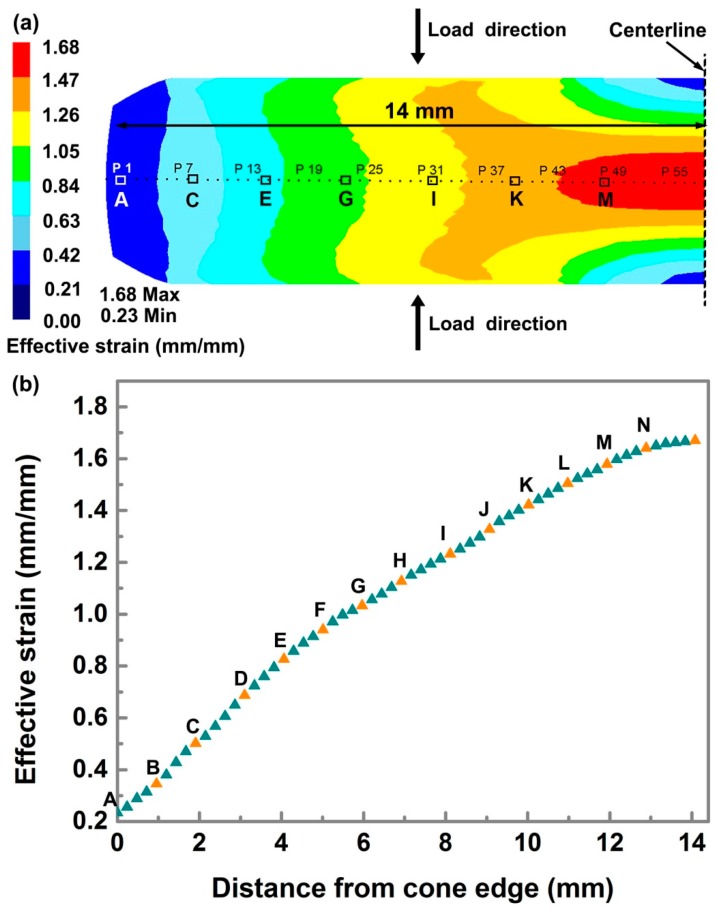
The strain distribution of the deformed specimen from the cone edge to the cone center after 70% reduction: (**a**) Illustration of simulation results corresponding to deformed specimen showing continuous distribution in effective strain ranging from 0.23 to 1.68 mm/mm; (**b**) Variation of effective strain with distance from the edge to the center of specimen, points A, B,…, N are subjected to microstructure examinations.

Figure 3 shows the strain distribution of the deformed specimen across the whole view after compressing to 70% reduction. It should be pointed out that only a quarter of the sample is presented here due to the symmetry of DCS. Continuous distribution of effective strain ranging from 0.23 to 1.68 mm/mm is observed from the cone edge to the center. The strain gradients are produced due to different reduction suffered for the specimen from the edge to the center. The contact surface was forced to be compressed once the compression process was initiated, leading to a larger effective strain found near the centerline. The tilted slope was then gradually subjected to deformation with deformation progressing, which would bring about strain gradient along the radial direction. 

### 3.3. Microstructure Developments Related to Strain Amount

**Figure 4:**
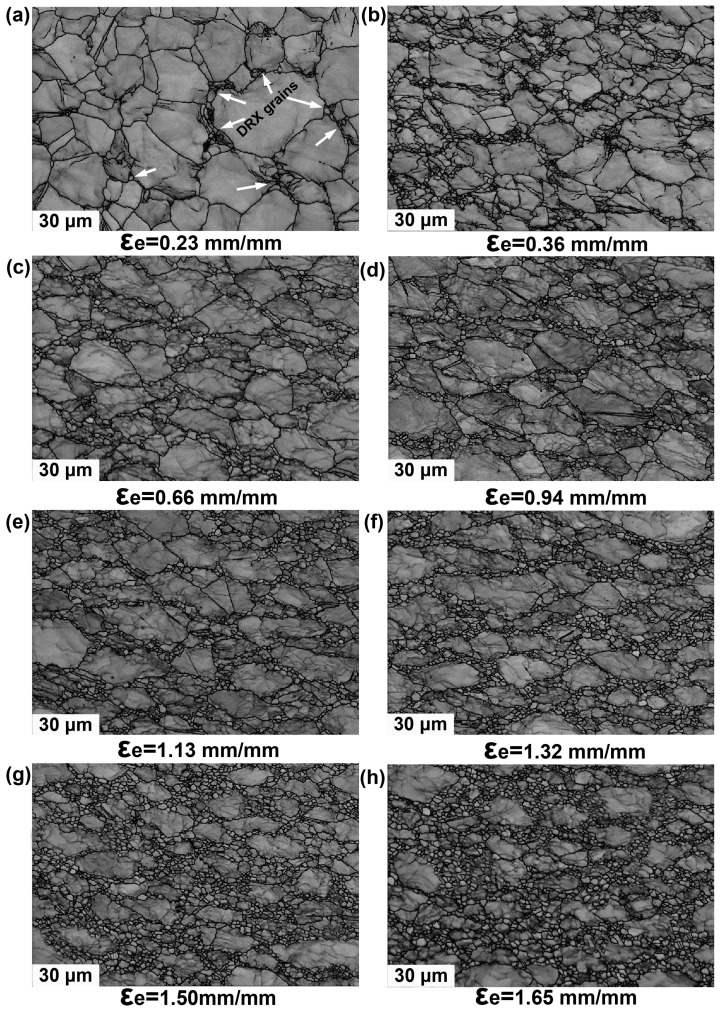
Strain amount dependent microstructure evolution corresponding to different location with various effective strains (mm/mm): (**a**) 0.23, showing the microstructure with new formed DRX grain of initial deformation period; (**b**) 0.36; (**c**) 0.66; (**d**) 0.94; (**e**) 1.13; (**f**) 1.32; (**g**) 1.50; (**h**) 1.65.

**Figure 5:**
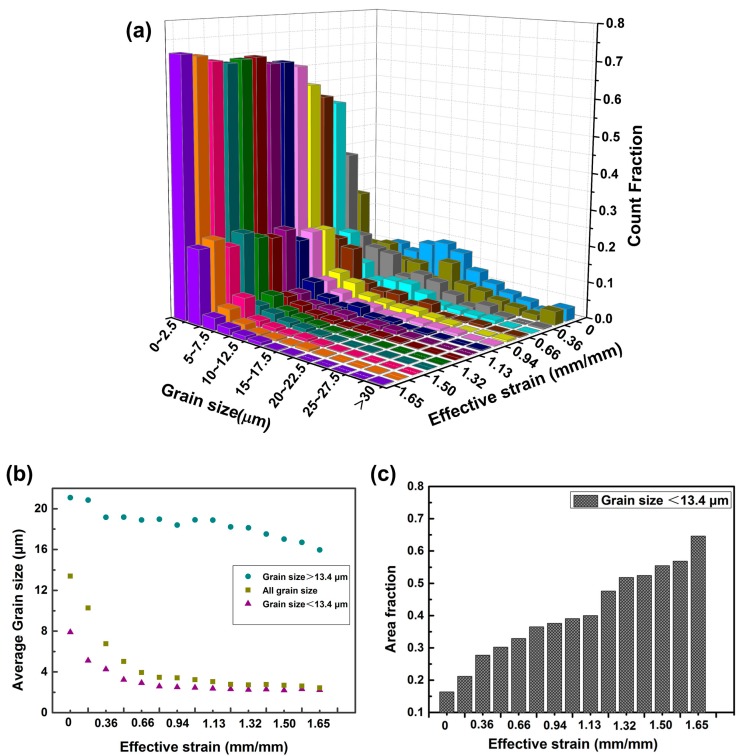
The grain size distribution and average grain size corresponding to different locations of deformed specimens: (**a**) The 3D grain size distribution corresponding to all the EBSD maps; (**b**) Average grain size for the groups with large grain size and fine grain size under different effective strain; (**c**) Area fraction of fine grains with a size below 13.4 μm under different effective strain.

EBSD examinations on point A to point N were carried out to investigate the effects of strain amount on microstructure and grain size evolutions. It should be noted that each marked point in Figure 3b is detected separately but only parts of EBSD maps out of 14 EBSD maps will be presented in the following discussions. As can be seen clearly in Figure 4, microstructure developments are significantly affected by strain mount, which is supposed to be dominated by DRX mechanisms during hot deformation. It is indicated from the band contrast maps the grain size is evidently refined with increasing strain amount. Figure 4a shows that a small fraction of grain boundaries become serrated at an effective strain of 0.23 mm/mm compared to the non-deformed grain boundaries. Moreover, a small amount of tiny grains are detected to be formed at the triple junctions, as indicated by white arrows. With strain increased to 0.36 mm/mm, the grains are slightly elongated which are accompanied with appearance of remarkable DRX grains, forming the proverbial ‘necklace-like’ structure. The new formed DRX grains will further grow up to reach nearly spherical shape with deformation continuing. After that, the DRX mechanism will occupy the dominant place in regulating the grain size evolution until accomplishment of deformation. Large grains are found to be refined gradually; meanwhile, more and more fine grains are generated next to each other with the increase of effective strain. The distribution of grain size and average grain size corresponding to specific positions are shown in Figure 5.

Figure 5a shows the 3D distribution of grain size corresponding to all the EBSD maps. In order to more clearly explore the grain size evolution, the distribution of initial grain size is also included in Figure 5a. The pristine microstructure corresponds to the case that effective strain is equal to zero. It is evident that once the effective strain reaches the critical strain for DRX, a remarkable number of refined grains emerges, leading the refinement of grain size. When the effective strain is increased to 0.36 mm/mm, the average grain size decreases dramatically to 6.76 μm from an initial average grain size of 13.4 μm. The average grain size reaches to a steady value about 2.5 μm after the effective strain is increased to1.22 mm/mm. Herein, to more accurately expound the contribution of DRX to the grain refinement, we artificially divide the grains into two groups: one is defined as grain size larger than 13.4 μm (group 1) and the other is smaller than 13.4 μm (group 2), based on the initial average grain size. The average grain sizes for both groups are determined and shown separately in Figure 5b. As can be seen clearly, the average grain size of group 1 keeps unchanged at the initial period of deformation. However, the average grain size of group 2 drops strikingly after the effective strain reaches a value of 0.33 owing to formation of considerable fine DRX grains, as is also validated in Figure 3 and Figure 4. With effective strain further increasing to 0.51 mm/mm, the average grain size of group 1 starts to decrease, accompanied with dramatic drop in overall average grain size. In this case, the ‘necklace-like’ structure is generated along the pristine grains, leading to the shrinkage of initial grains. After that, the average grain size of group 1 keeps steady fluctuations while the average grain size of group 2 is decreasing gradually. During the final period of deformation, a decrease in grain size of group 1 is observed, while keeping the overall average grain size stable. Physics behind the above phenomenon can be ascribed to the occurrence of remarkable DRX, which is accompanied by the growth of DRX grains, at the final period of deformation.

To quantitatively evaluate the refinement effect of DRX on grain size, the area fraction of fine grains is measured and shown in Figure 5c. Prior to deformation, the pristine microstructure shows a relatively low area fraction of fine grains (the grains with a size below the average grain size are defined as fine grains (FGs) in this article), which is down to 16.4%. The hypothesis is built here that the increase in area fraction of FGs is attributed to DRX progress. Under this premise, the large grains growing from FGs are not taken into account due to the sluggish grain growth process. It turned out that up to 19.7% of FGs are detected when the effective strain is reached 0.23 mm/mm, indicating that at least 3.3% of FGs resulted from DRX are formed. After initiation of DRX, the area fraction of FGs will be continuously increased until accomplishment of deformation. Increasing effective strain brings about the increase of area fraction of FGs. As a result, an area fraction of FGs up to 64.6% together with a count fraction as high as 99.5% is obtained, meaning that large numbers of pristine grains have finished the DRX process. While a count percentage about 0.5% of large grains remains, occupying an area fraction about 35.4%.

### 3.4. Grain Boundaries Evolutions Related to Strain Amount

**Figure 6:**
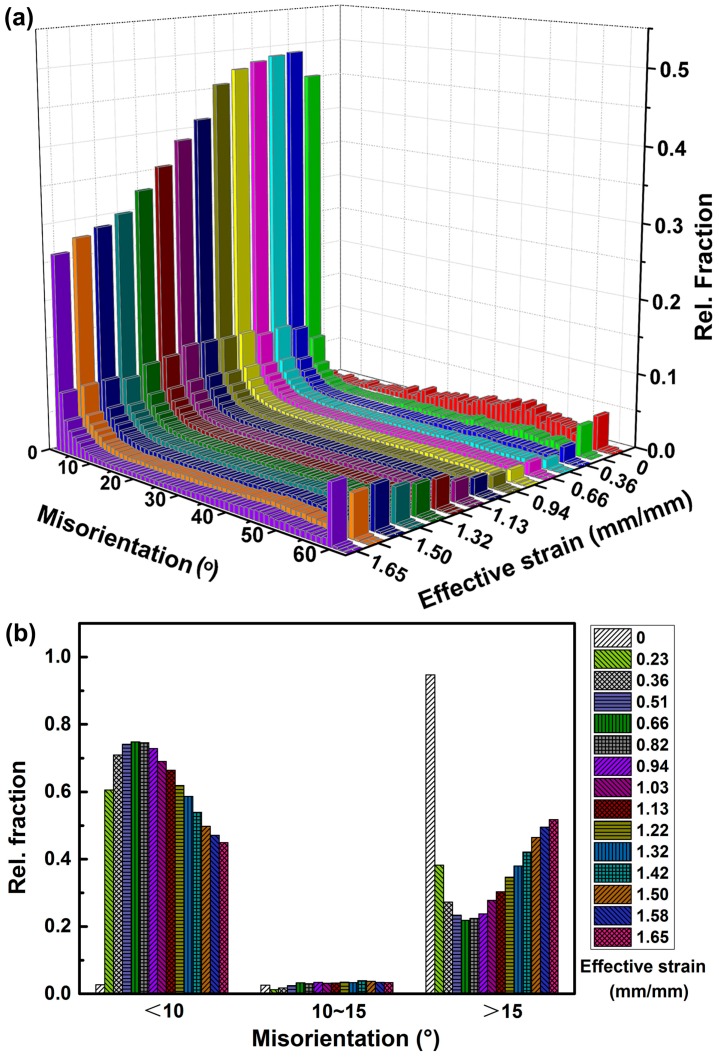
The boundary misorientations across the whole view of all 15 EBSD maps: (**a**) 3D misorientation distribution related to effective strain; (**b**) Relative fraction corresponding to different misorientation ranges, showing the variation tendency related to strain amount.

The boundary misorientations across the whole view for all 15 EBSD maps are measured to aid in further understanding of microstructure evolutions during hot compression. The boundaries with misorientation above 15° are generally supposed to be an indication of fully recrystallized grains, whilst boundaries with misorientation lower than 15° are typically related to sub-grain microstructures. As can be seen clearly from Figure 6a, prior to compression, as high as 94.0% boundaries belong to high angle grain boundaries. However, as deformation proceeds, a sharp rise up to 60% in LAGBs is observed at the effective strain of 0.23 mm/mm. The LAGBs will be transformed into HAGBs after initiation of DRX process. In spite of this, the LAGBs will be continuously generated with deformation going on, as shown in Figure 6a. Consequently, there will be a balanced point where the percentage of LAGBs arrives at the summit and the LAGBs start to decrease. After that, the HAGBs generated from DRX begin to increase gradually until the end of deformation. To demonstrate more clearly the relationship between grain boundary evolution and DRX progress, we artificially divide the LAGBs into two categories: one is that with misorientation lower than 10°(corresponding to sub-grains) and the other is between 10~15 ° (treated as transition zone from sub-grains to DRX grains) [26]. The corresponding relative fraction is summarized in Figure 6b. It is evident that the LAGBs tend to accumulate in the early stage of hot deformation, where the DRX is still sluggish. After initiation of significant DRX, new grains are formed in the vicinity of the deformed grain boundaries, where high density of LAGBs is generated, as shown in Figure 3. It should be pointed out that the fraction of misorientation angle between 10~15° keeps a steady and low level during the whole deformation process, indicating the rapid transition from LAGBs to HAGBs as well as DRX process.

**Figure 7:**
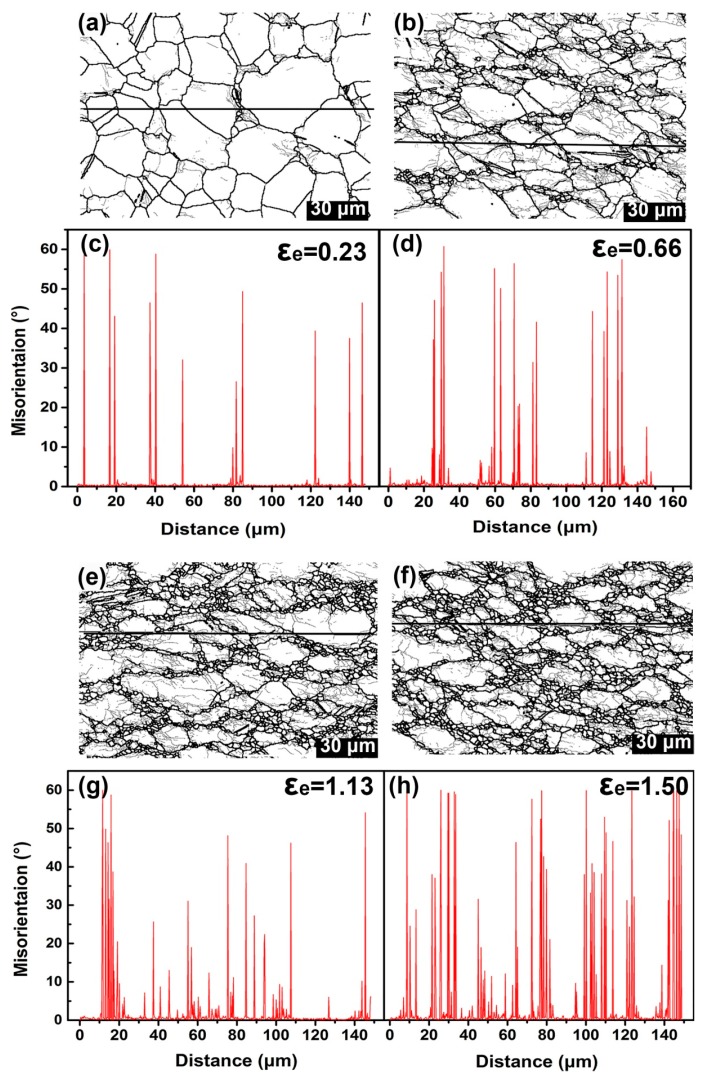
Misorientation developments corresponding to effective strain increase, showing the point-to-point misorientation for specific grains at different effective strain: (**a**, **b**) EBSD map showing the grain boundary characteristics at effective 0.23 and 0.66 mm/mm; (**c**, **d**) Distribution of point to point misorientation along the corresponded line drawn in EBSD map; (**e**, **f**) EBSD map showing the grain boundary characteristics at effective 1.13 and 1.50 mm/mm; (**g**, **h**) Distribution of point to point misorientation along the corresponding line drawn in EBSD map.

**Figure 8:**
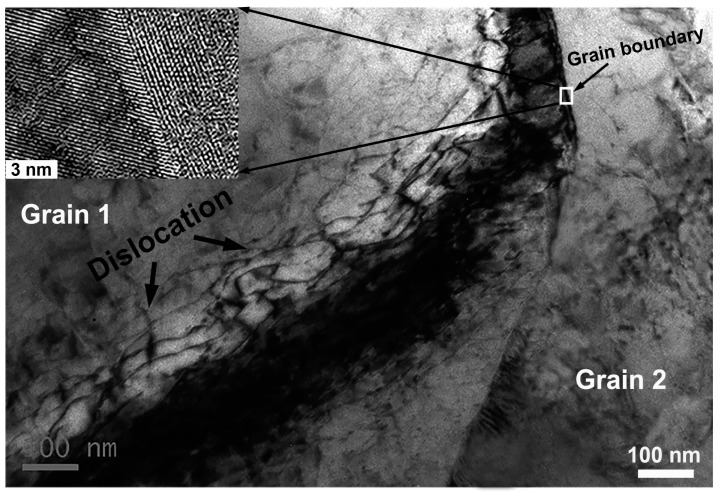
TEM observation showing the dislocation piled up at the grain boundary after small deformation. The high resolution image presented in the top left corner clearly demonstrates that a few atomic planes are distorted at different levels.

In order to shed more light on understanding the misorientation developments corresponding to effective strain increase, the orientation variations within a specific grain are analyzed and presented in Figure 7. The point-to-point misorientation of different grains along the line (marked by black lines in EBSD maps) is measured. As mentioned above in Figure 2c, prior to deformation, orientations within the single grain are very similar to each other, leading to an extremely low and stable misorientation across the whole grain. Once the deformation is produced, small misorientations within the grains, mostly near the grain boundaries, are seen to generate extensively. To figure out the microstructure of LAGBs, TEM examinations are employed here and shown in Figure 8. It is clearly demonstrated that the misorientations near the grain boundaries are manifested as the appearance of considerable dislocation after small deformation. The high-resolution image presented in the top left corner clearly demonstrates a few atomic planes are distorted at different levels. As deformation proceeds, the small misorientation near the grain boundaries will be developed into HAGBs by virtue of generating new grains driven by DRX, as show in Figure 7b. Measurements on misorientation variations demonstrate that HAGBs are continually shown in the vicinity of the grain boundaries in company with high fluctuation of misorientation within the grains, as shown in Figure 7d. Wherein, the continuously sharp increases of misorientation with grains are apparently observed, indicating formation of remarkable DRX grains. As deformation proceeds, more and more DRX grains are found to be generated near the grain boundaries, accompanied with formation of considerable HAGBs, as illustrated in Figure 7e–h.

**Figure 9:**
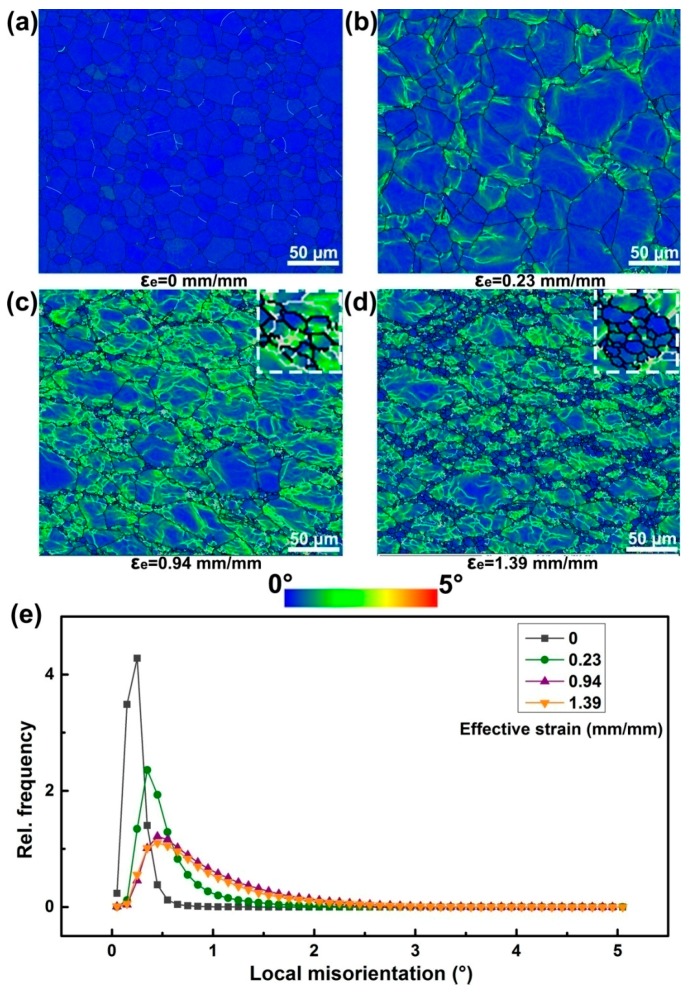
EBSD maps show the local misorientation mapping (ranging from 0~5°, marked by rainbow color, showing the higher deformation regions), grain boundaries (marked by black line) and subgrain boundaries (marked by silver line), corresponding to different effective strains: (**a**) pristine microstructure; (**b**) 0.23 mm/mm; (**c**) 0.94 mm/mm; (**d**) 1.39 mm/mm; (**e**) relative fraction and distribution.

To further bridge the relation among grain boundary behaviors, strain amount, and DRX process, the local misorientation mapping (LMM) for all EBSD maps are measurable. Herein, the LMM is employed to display small orientation changes within each grain and of the whole maps, highlighting the regions of higher deformation. The component calculates the average misorientation between every pixel and its surrounding pixels, and assigns the mean value to that pixel. Misorientations over 5° in the current work are discarded, so that the misorientations associated with discrete subgrains and grain boundaries are excluded. The selected LMM out of 15 EBSD maps are presented in Figure 9, showing the developments of local misorientation with increase of effective strain. Prior to deformation, the value of local misorientation shown in Figure 9a is observed to situate mostly in the range from 0~0.5°, indicating the nearly identical orientation of the whole scans. In other words, the value of local misorientation can be somehow related with the degree of deformation. Once the load is imposed, the grains are forced to deformation, leading to the formation of dislocation according to the above analysis. Accumulation of high dislocation density will produce misorientation in the highly deformed region. It is clearly found from Figure 9b that the higher deformation occurs mostly in the vicinity of grain boundaries, where the neonatal-fine grains are also observed. With effective strain increased to 0.94 mm/mm, a large amount of grains are more deformed, presenting a higher local misorientation near the grain boundaries. We add the grain boundaries on the maps and find that the subgrain boundaries are highly overlapped with the higher deformation region, as can be found more clearly from the close-up view in the top-right corner of Figure 9c. It is evident that the new formed grains contain a few subgrains, which will disappear gradually with deformation proceeding. The disappearing subgrains are supposed to be transferred into new grains with HAGBs, as it is indicated in Figure 9d, which is clearly observed in the macroscopic view at the top-right corner. It is apparently observed that abundant DRX grains with identical orientation are generated near the highly deformed region. In addition, the fraction of higher local misorientation is found to decrease with effective strain to 1.39 mm/mm where large amounts of DRX grains are simultaneously generated. This observation provides evidence to connect the transformation of subgrains with formation of DRX grains and shed more light on understanding why the new grains tend to generate at the grain boundaries with higher deformation.

Based on the above analyses, the DCS is experimentally proved to be an efficient mean in exploring and optimizing the hot deformation process. Using this high-throughput method, continuous strain gradients with wide ranges can be realized in a single specimen and single experiment. The techniques of EBSD and TEM are employed to aid in understanding the relationship among microstructure evolutions, grain size variations, orientation developments, and DRX progress with strain amount. Small misorientation tends to generate near the grain boundaries, which is manifested as piled-up dislocation in micromechanics. After the dislocation density reaches a critical value, DRX progress would be initiated at a higher deformation region, leading to the refinement of the grain size. During this process, the transformations from LAGBs to HAGBs and from subgrains to DRX grains are found to occur. After accomplishment of DRX progress, the neonatal grains are presented with similar orientation inside the grain boundary.

## 4. Conclusions

The microstructure development and grain boundary evolution of a nickel base superalloy during hot compression were investigated by introducing the high-throughput DCS. The following conclusions can be drawn based on the above analyses:

1. Continuous variations of effective strain ranging from 0.23 to 1.65 across the whole sample were achieved after reaching a reduction of 70%. Gradients of microstructure and grain size were found to exist from the edge to the center of the specimen.
2. Grain size was measured to be decreased with increase of effective strain form the edge to the center of specimen. The average grain size decreased dramatically at the initial deformation period and kept at a stable value by balancing the process of DRX and grain growth. The area fraction of fine grains was observed to increase all the way to the end of deformation due to successive DRX process.
3. The grain boundaries are the first sites to produce misorientation, which is manifested as the formation of dislocation. Subgrains are proved to be further generated in the higher deformation regions near the grain boundaries, where new DRX grains are inclined to be generated.

## Figures and Tables

**Table 1:** The nominal composition of the studied nickel base superalloy (wt. %).

Element	Co	Cr	Ti	W	Mo	Al	Nb	Hf	C	B	Zr	Ni
wt. %	26	13	3.7	4	4	3.2	0.95	0.2	0.05	0.025	0.05	Bal.
